# Decision Strategies in AI-Based Ensemble Models in Opportunistic Alzheimer’s Detection from Structural MRI

**DOI:** 10.1007/s10278-025-01604-5

**Published:** 2025-09-17

**Authors:** Solveig Kristina Hammonds, Trygve Eftestøl, Kathinka Daehli Kurz, Alvaro Fernandez-Quilez

**Affiliations:** 1 Stavanger Medical Imaging Laboratory, Radiology Department, Stavanger University Hospital, Gerd-Ragna Bloch Thorsens gate 8, Stavanger, 4011 Norway; 2 Centre for Age-Related Medicine (SESAM), Stavanger University Hospital, Gerd-Ragna Bloch Thorsens gate 8, Stavanger, 4011 Norway; 3 Department of Electrical Engineering and Computer Science, University of Stavanger, Kjølv Egelands hus, Kristine Bonnevies vei 22, Stavanger, 4021 Norway

**Keywords:** Alzheimer’s disease, MRI, Ensemble model, Deep learning, Calibration

## Abstract

Alzheimer’s disease (AD) is a neurodegenerative condition and the most common form of dementia. Recent developments in AD treatment call for robust diagnostic tools to facilitate medical decision-making. Despite progress for early diagnostic tests, there remains uncertainty about clinical use. Structural magnetic resonance imaging (MRI), as a readily available imaging tool in the current AD diagnostic pathway, in combination with artificial intelligence, offers opportunities of added value beyond symptomatic evaluation. However, MRI studies in AD tend to suffer from small datasets and consequently limited generalizability. Although ensemble models take advantage of the strengths of several models to improve performance and generalizability, there is little knowledge of how the different ensemble models compare performance-wise and the relationship between detection performance and model calibration. The latter is especially relevant for clinical translatability. In our study, we applied three ensemble decision strategies with three different deep learning architectures for multi-class AD detection with structural MRI. For two of the three architectures, the weighted average was the best decision strategy in terms of balanced accuracy and calibration error. In contrast to the base models, the results of the ensemble models showed that the best detection performance corresponded to the lowest calibration error, independent of the architecture. For each architecture, the best ensemble model reduced the estimated calibration error compared to the base model average from (1) 0.174±0.01 to 0.164±0.04, (2) 0.182±0.02 to 0.141±0.04, and (3) 0.269±0.08 to 0.240±0.04 and increased the balanced accuracy from (1) 0.527±0.05 to 0.608±0.06, (2) 0.417±0.03 to 0.456±0.04, and (3) 0.348±0.02 to 0.371±0.03.

## Introduction

Alzheimer’s disease (AD) is a neurodegenerative disease that causes irreversible damage to brain tissue and accounts for 60–70% of cases of dementia worldwide [1]. The disease is characterized by accumulation of neurofibrillary tangles and amyloid plaques that are specific biomarkers of AD [2]. As AD develops, clinical symptoms such as cognitive impairment, disruption of activities of daily living, and behavioral changes can be observed, but neurodegeneration cannot be reversed. In light of recent developments in medical interventions that target amyloid plaque build-up in the brain [3], early confirmation of neurodegenerative decline leading to AD can make a difference in future individual treatment plans. The choice of treatment will depend on individual variations in the expression and origin of the disease. This requires knowledge of the individual biomarker signature, which neuroimaging can contribute together with fluid biomarkers. Amyloid positron emission tomography (PET) is a preferred neuroimaging modality for early assessment of AD biomarkers, while structural magnetic resonance imaging (MRI) is a common supplement in the current standard diagnostic workup for dementia and is widely used in dementia research [4, 5]. Unfortunately, the availability of amyloid and tau PET for clinical use is still limited, and blood-based biomarkers still require standardization [6, 7], while structural MRI is already a widely available imaging modality in the clinic today. This role is likely to be strengthened considering the necessary monitoring of the side effects of anti-amyloid treatment [8]. However, the clinical value of structural MRI is still held back by its current application, which requires human interpretation. Therefore, the findings are limited to structural changes visible to the human eye, which are known to be detectable only later in the AD trajectory when the consequences of neuronal changes are visible.

There is a growing trend for decisions to be supported by artificial intelligence (AI) in all areas of medicine, including dementia. Commonly referred uses of AI for AD are the selection of eligible candidates for medical interventions, the early identification of disease risk, and the improved analysis of medical images with a lower human work burden [9]. With AI methods, there is the potential to extract value and combine data in a way humans alone cannot, opening up to the opportunistic detection of AD using structural MRI in combination with AI. However, deep learning (DL) imaging projects in AD often suffer from small dataset sizes, limited representation of atypical cases in training data, selection bias, and reporting bias, which has consequences for both the generalizability of the results and translation to clinical utility [10, 11]. In this context, good detection and calibration are desirable, but the latter is typically neglected. Calibration performance identifies how closely the estimated prediction probability aligns with the true representation. Failing to address model calibration for AI models before clinical application could have consequences for diagnostics and treatment plans in terms of under- and overdiagnosis, as well as under- and overtreatment [12].

Ensemble models that combine results from several models have the ability to manage the risk of overfitting and underfitting that is associated with variance and bias of single DL models, take advantage of the diversity between models, and offer increased generalizability of the models to new data. Boosting, stacking, and bagging are commonly used types of ensemble technique [13]. Stacking techniques typically achieve diversity by combining predictions from different learning architectures through an additional learning step. Boosting techniques are usually characterized by sequential learning and weighting, where one model attempts to correct the misclassifications of the previous model, and as such tend to focus on the examples that are most difficult to predict. Bagging is a type of parallel ensemble that originates from bootstrap aggregation [14]. In general, bagging methods achieve diversity between base models by applying the same algorithm independently to several overlapping subsamples of the total training. Parallel ensemble models offer a simple way to combine predictions from individual base models. One of their main strengths is to reduce variance, which is associated with overfitting [15, 16]. The combination of prediction results from several models requires a strategy to determine the final prediction label. The majority vote, the unweighted and the weighted average are examples of common decision strategies used for ensemble models in medical imaging. Combining a number of weaker classifiers can improve the final detection performance, and ensemble models can help reduce the statistical and representational problems that small datasets often suffer from [15]. Although the role of ensemble models for improved detection is well known, the interplay between their calibration and detection ability is less explored [17].

The purpose of our study is to analyze the relationship between detection performance and calibration for decision strategies in DL-based ensemble methods using structural MRI. We limit our work to bagging and related ensemble methods in which base models are trained in parallel using a subset of the original training data and combined at the decision level to derive the final prediction. The main contributions of our study are as follows:

- We employ detection metrics that consider class imbalance and are more representative of expected clinical performance,
- We train and evaluate three different ensemble decision strategies for AD detection; including development of a state-of-the-art weighted ensemble decision strategy,
- We measure the effect of the ensemble decision strategies on calibration using estimated calibration error (ECE) and reliability diagrams,
- We explore the generalization of the results from the perspective of three different deep learning classification architectures.

The article is structured as follows: Section 2 presents relevant related work in the field of dementia. Section 3 provides detailed information about materials and methods to allow others to replicate and verify the results. Section 4 describes the results. Sections 5 and 6 discuss and conclude the findings, limitations, and future work.

**Table 1:** An overview of related work presenting deep learning classification of Alzheimer’s disease (AD) from structural brain magnetic resonance imaging (MRI) using bagging-type ensemble methods

Authors	Classification	Ensemble	Decision	Evaluation	Result
		architectures	strategy	metric	
Gawade et al. [27]	*Multi-class*:	LeNet + UNet	Weighted	Accuracy	0.967
	non-demented		average		
	very mild AD				
	mild AD				
	moderate AD				
Mofrad et al. [22]	*Binary*:	ResNet-18	Majority vote	Accuracy	0.759
	stable MCI	+ ResNet34		Weighted	
	converting MCI			precision	0.771
				F1 score	0.759
			Unweighted	Accuracy	0.763
			average	Weighted	
				precision	0.775
				F1 score	0.766
Simone et al. [31]	*Binary*:	Custom	Weighted	Accuracy	0.731
	CN	3D CNN	average	ROCAUC	0.809
	MCI/AD	(VGG-style)			
	*Multi-class*:	Note:		Accuracy	0.831
	CN	Multimodal		ROCAUC	0.882
	MCI	MRI + PET			
	AD				
Mercaldo et al. [30]	*Multi-class:*	Custom	Unweighted	Accuracy	0.952
	CN	2D CNN	average	Precision	0.953
	MCI	(Inception-		Sensitivity	0.950
	AD	style)		ROCAUC	0.987
Leming et al. [29]	*Binary:*	ResNet-50 (3D)	Unweighted	ROCAUC	0.859
	CN		average		(structural
	MCI/AD				3D MRI)
Ma et al. [28]	*Multi-class:*	MLP	Unweighted	Accuracy	0.868
	CN		average		
	AD				
	FTD				
Qui et al. [23]	*Multi-class:*	DenseNet +	Unweighted	Accuracy	0.926
	CN	Inception-V4	average		
	MCI				
	AD				
Islam et al. [18]	*Multi-class:*	Custom	Majority vote	Accuracy	0.932
	non-demented	2D CNN		Precision	0.94
	very mild AD	(DenseNet-		Sensitivity	0.93
	mild AD	style)		F1 score	0.92
	moderate AD				

CN, cognitively normal; CNN, convolutional neural network; FTD, fronto-temporal dementia; MCI, mild cognitive impairment; MLP, multi-layer perceptron; PET, positron emission tomography; ROCAUC, area under the receiver operating characteristics curve

## Related work

An overview of related work is presented in Table 1. To address the use of small imbalanced datasets for AD classification, Islam et al. [18] trained five base models with similar architectures based on DenseNet principles [19] with four dense blocks interleaved by transition layers, varying only the number of filter kernels in the last two dense blocks. Their proposed majority vote ensemble model for the prediction of four cognitive states (non-demented, very mild dementia, mild dementia, and moderate dementia) from structural MRI combined the three best-performing base models. The proposed ensemble model outperformed two other majority vote ensemble models consisting, respectively, of four and five of the base models, and two baseline models trained with the Inception-v4 [20] and ResNet [21] architectures. To compensate for the underrepresentation of the less frequent classes, the loss functions were modified using a class-dependent cost matrix.

Majority vote and unweighted average ensembles were applied by Mofrad et al. in a solution that converts longitudinal image-derived measurements into 2D images to allow the use of standard image classification methods with longitudinal data to discriminate between stable mild cognitive impairment (MCI) and MCI that converts to AD [22]. The proposed method, which can be applied to imbalanced data sets and allows missing data, showed promising classification results for cognitive states that are difficult to discriminate. The ensemble models consisting of base models of the ResNet-18 and ResNet-34 architectures with slight variations of dropout probability, weight decay, and maximum learning rate, improved classification performance compared to most individual base models measured by the area under the receiver operating curve (ROC AUC). The choice of decision strategies appears to be insignificant in this experiment, as both types of ensemble had similar accuracy, weighted precision, recall, and F1 results.

Among the few weighted average strategies we found that was used for AD detection, Qui et al. [23] proposed a two-step weighting approach for a multi-class detection of cognitively normal (CN), MCI and AD using an ensemble of DenseNet and Inception-v4, both pre-trained with the ImageNet dataset [24]. For both architectures separately, the first step normalized each sample’s softmax probabilities to the maximum of the three class probabilities. In the next step, the normalized class detections for the two architectures were combined by sample-wise vector multiplication, and then normalized again. The highest class probability for each sample determined the final predicted label. This setup improved the individual accuracies of DenseNet and Inception-v4 of 0.912 and 0.824, respectively, to an accuracy of 0.926 for the weighted average ensemble. A simpler weighted average ensemble approach based on the LeNet [25] and U-Net [26] architectures determined the weights from the accuracies of the base models [27]. The authors of this ensemble model hypothesized that the networks were originally designed for different imaging tasks and would therefore provide different feature maps during training, which would strengthen an ensemble of the two.

Unweighted average ensemble models that combined 10 DL models trained from 10 subsets of the training data, in a manner similar to cross-validation, increased the robustness of the model for a task of differentiating between fronto-temporal dementia, AD, and subjects with normal cognition using structural MRI [28]. Here, the neural network was constructed of multi-layer perceptrons (MLP). The ensemble models improved results compared to individual base models in three of the four experiments, where volume and thickness features were acquired from patches of different scales. In a fourth experiment, a generative adversarial network was applied as an alternative to augmentation operations such as rotation, flipping, and zooming that are commonly used with 2D and 3D image patches. In this experiment, the GAN itself demonstrated better stability between individual base models compared to previous experiments. For this reason and to reduce computational expense, the authors suggested that the use of ensemble classifier could be considered optional for the models trained with GAN. Two other examples of unweighted average ensemble models improved model performance for a binary classification between dementia and normal cognition after constructing a confounder-free image database that incorporated a combination of MRI modalities [29] and combining predictions based on individual image slice directions from structural MRI to discriminate between CN, MCI, and AD [30].

Ensemble learning has been suggested as a suitable method for combining data from multiple modalities. The process of training the data modalities separately and assembling the results from the base models was compared to concatenating the data prior to training the model in a recent study that combined structural MRI with metabolic activity data from positron emission tomography [31]. They showed that the ensemble model drastically improved the results compared to the base model trained on MRI data alone and that both modalities benefited from the ensemble with respect to detecting the AD class. It is worth mentioning an ensemble model consisting of two DL models trained on 2D axial slices from structural MRI and a custom model trained on clinical data that outperformed several recent state-of-the-art models for detecting AD with binary classification of AD/CN, MCI/CN and MCI/AD [32]. The outputs of the three base models were concatenated and passed through two fully connected layers where the final predictions were determined by the softmax function. It is notable that the inclusion of clinical data had a considerable effect on the performance of the ensemble model pushing the accuracy from 68.85 to 93.44% when combined with only one DL model, and to 99.67% when combined with both DL models.

**Fig. 1:**
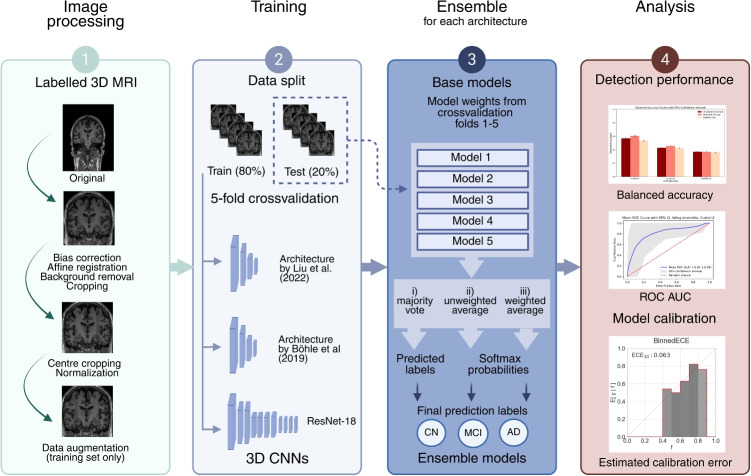
Overview of the workflow. AD, Alzheimer’s disease, CNN, convolutional neural network, CN, cognitively normal, MCI, mild cognitive impairment, MRI, magnetic resonance imaging

With respect to detection performance, the choice of performance metrics and how we can interpret the results are affected by the study design, for example, defining the question as a binary problem vs. a multi-class problem, or balancing the data with respect to classes or features that are important for the research question [16, 33]. Interestingly, Guo et al. [34] showed that increased layer depth and width, batch normalization, and less weight decay, which have contributed to a significant increase in classification performance for convolutional neural networks compared to earlier machine learning models and simpler neural networks, at the same time contribute to increased model miscalibration. Model calibration in terms of ECE has its origin in forecasting [35], but has been explored and adapted in recent times for wider use within machine learning for binary and multi-class settings [34, 36]. In a recent paper, Dang et al. [37] measured fairness-related miscalibration reported as ECE in a MRI-based AD detection study using a state-of-the-art 3D convolutional neural network (CNN). The CNN consists of 5 convolutional blocks of convolutional, batch normalization, ReLU activation, and max pooling layers followed by three fully connected layers. The same architecture was originally deduced from a number of experiments to optimize the best architecture based on several published state-of-the-art CNNs for detection of AD with structural MRI. Dang et al. trained the model with fivefold cross-validation using CN, MCI, and AD subjects from the ADNI1, ADNI2, and ADNI-GO studies, but tested the model in a binary experiment with CN and AD subjects only. Their analyses of detection vs. calibration performance revealed severe calibration biases related to subgroups of sex, educational level, age, ethnicity, and genetic risk factors.

## Materials and Methods

### Data

A complete overview of the workflow of our study is presented in Fig. 1, with further details elaborated in the following sections.

#### Data Acquisition and Preprocessing

Data used in the preparation of this article were obtained from the Alzheimer’s Disease Neuroimaging Initiative (ADNI) database (adni.loni.usc.edu). As such, the investigators within the ADNI contributed to the design and implementation of ADNI and/or provided data but did not participate in analysis or writing of this report. A complete listing of ADNI investigators can be found at http://adni.loni.usc.edu/wp-content/uploads/how_to_apply/ADNI_Acknowledgement_List.pdf. The ADNI was launched in 2003 as a public-private partnership, led by Principal Investigator Michael W. Weiner, MD. The primary goal of ADNI has been to test whether serial MRI, PET, other biological markers, and clinical and neuropsychological assessment can be combined to measure the progression of MCI and early AD. For up-to-date information, see www.adni-info.org.

**Fig. 2:**
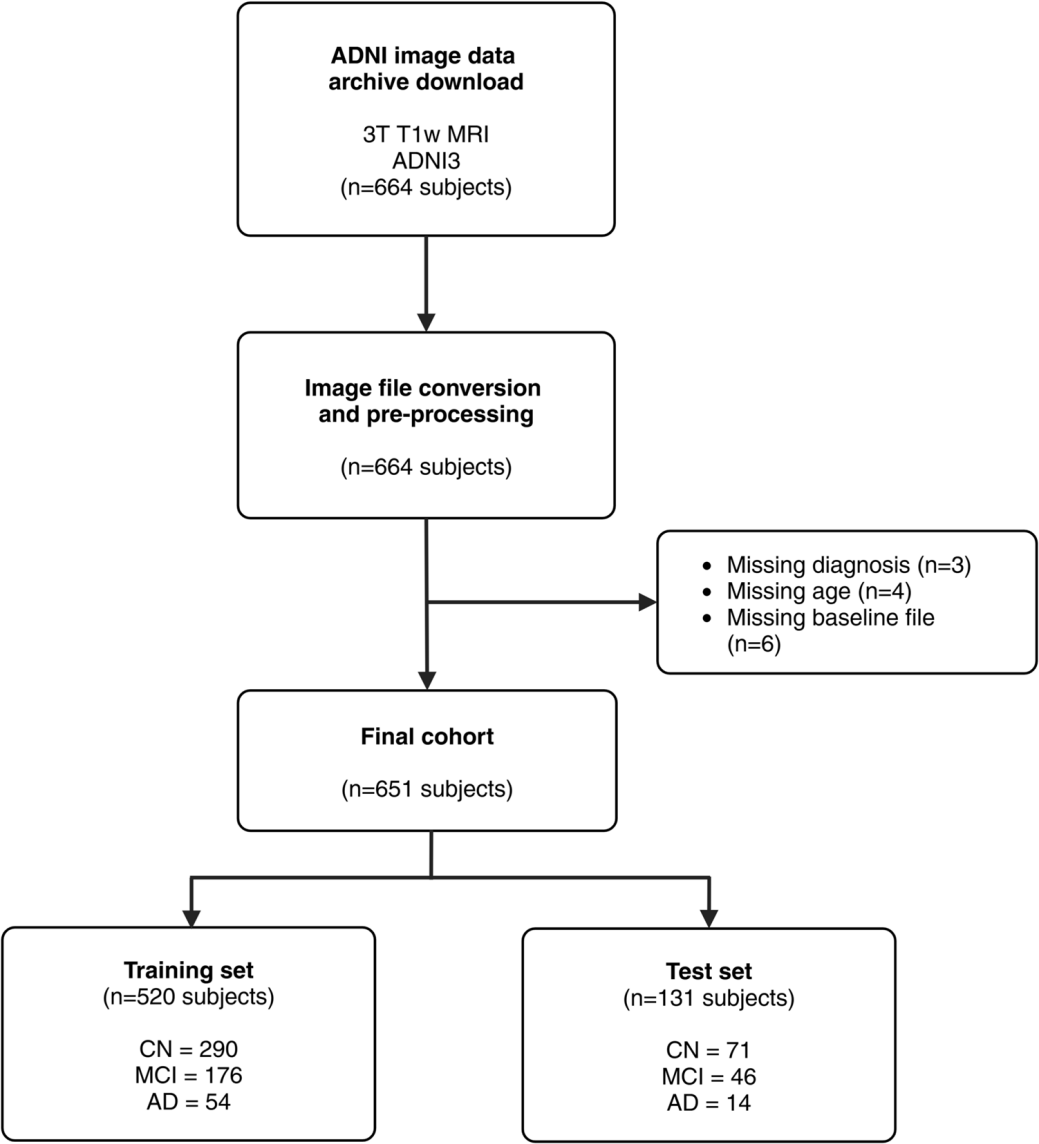
Population included in the study after applying inclusion and exclusion criteria. *AD*, Alzheimer’s disease; *CN*, cognitively normal; *MCI*, mild cognitive impairment

The clinical study information and image data were extracted from the study database 19 December 2022 by limiting the image search to “original image type” for “modality MRI,” “field strength 3,” and “weighting T1.” Inclusion was limited to baseline MRI and baseline clinical assessment results from newly enrolled participants of the ADNI3 study phase. An overview of the subject inclusion is presented in Fig. 2. The study population consists of three diagnostic categories: CN, MCI, and mild dementia caused by AD.

Full 3D volume structural MR images from the T1-weighted sequence magnetization prepared rapid gradient-echo MP-RAGE were used. Details of the ADNI3 MRI imaging protocol are available at https://adni.loni.usc.edu/wp-content/themes/freshnews-dev-v2/documents/mri/ADNI3-MRI-protocols.pdf. Deviations from the protocol with respect to the stated field-of-view (FOV) of 208 $\times$ 240 $\times$ 256 mm were handled during image preprocessing.

Two Clinica pipelines were used for data preprocessing [38]. The “adni-to-bids” pipeline converts images from original Digital Imaging and Communications in Medicine (DICOM) format to Neuroimaging Informatics Technology Initiative (NIfTI) format, organizes the files in Brain Imaging Data Structure (BIDS), excludes poor quality images, and produces a participants data file with demographic data and clinical test results. Demographic data and cognitive test results were extracted from the participants file, while diagnostic category per session was extracted from the DXSUM_PDXCONV_ADNIALL.csv file of the ADNI study information. The screening diagnosis was used for subjects for those cases where only screening visit diagnosis was available. Linear processing of the anatomical MR images was applied using the “t1-linear” pipeline that provided bias correction with N4ITK, affine registration to the MNI152NLin2009cSym template, and background removal by cropping to a matrix size of 169 $\times$ 208 $\times$ 179 with 1 mm isotropic voxels, as described in [38]. As part of our DL pipeline, images were further center-cropped to matrix size 160 $\times$ 160 $\times$ 160 and the voxel values normalized to values between 0 and 1.

#### Study Population and Ground Truth Labels

The ADNI3 study consists of newly recruited participants and participants rolled over from previous ADNI study phases. We included only newly recruited participants in order to restrict the image selection to baseline images acquired with the same field strength and under the same post-processing conditions. The inclusion and exclusion criteria are thoroughly described in the ADNI3 study protocol found on the ADNI website. According to the study protocols, men and women in the age range of 55 to 90 years were recruited for ADNI3 and distributed into three diagnostic classes: CN, MCI, and AD. The baseline diagnostic category was used for the ground-truth labeling of the images. The study’s selection into diagnostic categories is based on a number of criteria including subjective memory complaints, memory function documented by education adjusted cutoffs on the Logical Memory II subscale, Mini-Mental State Exam Score (MMSE), Clinical Dementia Rating (CDR), general cognition and functional performance, stability of medication, evaluation of absence of significant neurological disease other than AD, good general health, and geriatric depression scale score less than 6. The study protocol describes the priority order for all types of assessment in the event of insufficient time to complete the full visit, which means that some participants have missing values for some of the assessments.

### Neural Network Architectures

We performed three ensemble experiments, each with a different neural network architecture, to examine the consistency of the results between architectures. ResNet is a classical neural network architecture that was developed for image recognition [21]. We used a 3D adaption of the original 2D ResNet-18 as a benchmark for two customized 3D neural networks [40], which will be addressed as Custom1 and Custom2. Both customized networks have characteristics of the VGG architecture [41] and have previously shown state-of-the-art performance in dementia detection using 3D structural MRI. Custom1 is described by Liu et al. [42] and was used for multi-class detection of CN, MCI, and AD from 3D structural MRI using data from both the ADNI study and the North American Alzheimer’s dataset from The National Alzheimer’s Coordinating Center. Custom2 was first described by Böhle et al. [43] for binary classification between CN and AD from 3D structural 1.5T MRI from the ADNI study. Later, Klingenberg et al. used the same architecture for binary classification between CN and AD from 3D structural 3T MRI [39]. Both customized networks are available from Github and were coded in Pytorch, which we translated into Keras versions for use in our pipeline.

### Model Training and Evaluation

The data were split at subject-level into a training set (80%) and a test set (20%) as shown in Fig. 2. Stratification by diagnostic category, sex, and age was applied to maintain the original class distribution and reduce vulnerability to some of the common features associated with dementia risk. The population was divided into age bins using 5-year ranges similar to [39], with the lower and upper age bins being open-ended to minimum and maximum age in order to ensure a sufficient number of participants in each group. We applied age bins: age1 ($\le 64$ years), age2 (65–69 years), age3 (70–74 years), age4 (75–80 years), and age5 ($\ge$81 years).

The models were trained by applying five-fold cross-validation to the training set. The standard data augmentation techniques flip and translate were used for images in the training set for the purpose of adding data variability. Random sagittal flip with a probability of 0.5 and random sagittal translation in the range of (-3,3) were applied to the images. Detection performance was evaluated with the test set using several performance metrics. To account for class imbalance of the data set, balanced accuracy and weighted precision, weighted recall (sensitivity), weighted specificity, and area under the receiver operating characteristic curve (ROC AUC) one-vs.-one were used. The latter calculates the average AUC for all pairwise class combinations. An NVIDIA CUDA parallel computing platform with AMD EPYC 7302 CPU @ 3 GHz nodes with 3 NVIDIA A100 SXM4 40GB GPUs and Intel Xeon 4214R CPU @2.4 GHz nodes with 2 NVIDIA A100 PCIE 40GB GPUs was used for training and evaluation of all model architectures.

### Ensemble Decision Strategies

Generally, the decision methods used in ensembles are variations of majority voting and averaging that can either be weighted or unweighted. For our study, we explore and compare the use of standard majority voting without weighting, an unweighted average method, and a type of weighted average method to predict the final diagnostic class for each image of the test set. In the case of “majority voting,” a number of discrete class labels are determined by counting the number of label instances for each class provided by the individual base models and assigning the final label to the class with most votes. The labels of each base model are determined by the class labels of the softmax class with the highest (pseudo) probabilities. Our majority vote ensembles combine the hard labels, not the underlying probabilities of each base model. We use the term “unweighted average” to refer to the combination of the estimated softmax probabilities for each class from a number of base models and determine the final label based on the highest combined probability. All base models are assigned the same weight. Similarly, by “weighted average,” we refer to methods that assign different weights to different base models before the softmax class probabilities are combined and the final label is concluded. This can be compared to varying the priority of suggestions from specific members of an expert group.

**Table 2:** Descriptive characteristics of the study population

	All	CN	MCI	AD	Statistics
**Participants**					
Total, *n*	651	361	222	68	
Male:female	295:353	132:229	122:100	41:27	$\chi ^2$(2)=25.64, p $<0.001$
Female, %	54.2	63.4	45.0	39.70	
**Age**					
Years	70.83 ±7.34	69.50 ±6.74	71.91 ±7.57	74.37 ±7.94	$\chi ^2$(2)=25.24, *p* $<0.001$
Age1 ($\le$64 y), *n*	119	64	47	8	
Age2 (65–69 y), *n*	196	151	35	10	$\chi ^2$(8)=64.80
Age3 (70–74 y), *n*	142	72	54	16	$p <0.001$
Age4 (75–80 y), *n*	125	46	62	17	
Age5 ($\ge$81 y), *n*	69	28	24	17	
**Education**					
Years	16.43 ±2.32	16.69 ±2.22	16.26 ±2.39	15.62 ±2.42	$\chi ^2$(2)=13.00, p $<0.05$
Cognitive evaluations					
MMSE	27.52 ±2.93	29.07 ±1.18	27.59 ±2.07	22.90 ±2.75	$\chi ^2$(2)=232.36, $p <0.001$
MoCA	24.03 ±4.20	26.06 ±2.64	22.82 ±3.29	16.88 ±4.41	$\chi ^2$(2)=242.92, $p <0.001$
Missing, *n*	19	6	10	3	
ADAS-Cog13	13.04 ±8.81	8.30 ±4.29	15.36 ±6.6	30.39 ±7.95	$\chi ^2$(2)=297.94, p $<0.001$
Missing, *n*	6	4	2	0	
**APOE status**					
APOE4 negative 0 allele, n	278	182	81	15	$\chi ^2$(2)=25.04, p $<0.001*$
APOE4 positive 1 / 2 allele, n	160 / 37	85 / 9	51 / 15	24 / 13	*neg vs pos
Missing, *n*	176	85	75	16	Missing ignored

Continuous variables are presented as mean ± standard deviation. Kruskal-Wallis test statistics were used for continuous variable comparison between diagnostic groups, while chi-square were used for categorical variable comparison. Both tests are report as $\chi ^2$(degrees of freedom). The table includes the distribution of participants per age bin, which was used as stratification criteria for the splitting of datasets. For ages bins, additional Spearman’s rank-order correlation coefficient was calculated for CN/MCI ($\rho$=0.10, *p*=0.873), CN/AD ($\rho$=$-$0.67, *p*=0.219), and MCI/AD ($\rho$=0.10, *p*=0.870) showing no pairwise correlation. A *p*-value $< 0.05$ is considered significant. AD, mild Alzheimer’s disease; ADAS-Cog13, Alzheimer’s Disease Assessment Scale - Cognitive Subscale version 13; APOE4, Apolipoprotein E4; CN, cognitively normal; MCI, mild cognitive impairment; MMSE, Mini-Mental State Exam Score; MoCA, Montreal Cognitive Assessment

#### Weighting Strategy

We modify a weighting solution from Iqball and Wani [44] who defined the weights based on the accuracies of the base models from training validation. The multiplication factor $\alpha$ for each model was calculated by subtracting the lowest accuracy value of the base models from the accuracy value of each model. To ensure that the lowest-performing model in terms of accuracy will contribute to the ensemble, a constant of 1 was added. Due to the class imbalance of our dataset, we replace accuracy with balanced accuracy in our modification of the weighting strategy of Iqball and Wani [44]. To add distance between the models and prioritize the strongest performing models, we propose that the models are ranked from 1 to *m* (*m* = number of base models) with lower values assigned to weaker performing models, and that the multiplication factor $\alpha _{j}$ is calculated by multiplying the original multiplication factor of Iqball and Wani [44] by the square of the rank number. The final weights $w_j$ are determined by normalizing the multiplication factor $\alpha _{j}$:

1 $$\begin{aligned} \alpha _{j} = (\text {bacc}_{j} - \beta + 1) \times \text {rank}_{j}^2, \quad j = 1, \ldots , m \end{aligned}$$

2 $$\begin{aligned} \beta = \min \left( \left[ \text {bacc}_{j} \right] _{j=1}^m \right) \end{aligned}$$

3 $$\begin{aligned} w_{j} = \frac{\alpha _{j}}{\sum _{j=1}^{m} \alpha _{j}} \end{aligned}$$

Subscript *j* denotes the model number where m is the total number of models in the ensemble and bacc denotes balanced accuracy. In our experiment, *m* equals 5.

**Table 3:** General classification performance and estimated calibration error (ECE) of the base models for each architecture

Base model	Balanced accuracy		ROC AUC		ECE	
	Mean	95% CI	Mean	95% CI	Mean	95% CI
**Custom 1**						
Model 1	0.489	[0.479,0.499]***	0.723	[0.714,0.731]*	0.168	[0.159,0.176]$^{\text {ns}}$
Model 2	0.567	[0.555,0.578]***	0.703	[0.694,0.711]***	0.168	[0.161, 0.176]$^{\text {ns}}$
Model 3	0.487	[0.478,0.496]***	0.719	[0.711,0.727]**	0.179	[0.171,0.187]*
Model 4	0.491	[0.481,0.501]***	0.724	[0.716,0.732]*	**0**.**163**	[0.156,0.171]$^{\text {ref}}$
Model 5	**0**.**602**	[0.592,0.613]$^{\text {ref}}$	**0**.**738**	[0.728,0.748]$^{\text {ref}}$	0.192	[0.184,0.199]***
Average of means	0.527		0.721		0.174	
**Custom 2**						
Model 1	0.405	[0.397,0.413]***	0.739	[0.733,0.745]$^{\text {ns}}$	0.202	[0.195,0.210]***
Model 2	0.425	[0.422,0.438]***	**0**.**746**	[0.740,0.752]$^{\text {ref}}$	**0**.**147**	[0.139,0.155]$^{\text {ref}}$
Model 3	**0**.**473**	[0.464,0.483]$^{\text {ref}}$	0.742	[0.735,0.749]$^{\text {ns}}$	0.165	[0.159,0.172]***
Model 4	0.371	[0.367,0.375]***	0.718	[0.712,0.723]***	0.209	[0.202,0.216]***
Model 5	0.414	[0.409,0.420]***	0.739	[0.733,0.745]$^{\text {ns}}$	0.189	[0.182,0.196]***
Average of means	0.417		0.737		0.183	
**ResNet-18**						
Model 1	0.333	[0.333,0.333]***	0.535	[0.527,0.544]***	**0**.**165**	[0.157,0.172]$^{\text {ref}}$
Model 2	0.333	[0.333,0.333]***	0.545	[0.536,0.553]***	0.193	[0.157,0.172]***
Model 3	**0**.**370**	[0.362,0.378]$^{\text {ref}}$	0.649	[0.642,0.656]***	0.301	[0.185,0.201]***
Model 4	0.332	[0.328,0.337]***	0.625	[0.619,0.631]***	0.314	[0.305,0.322]***
Model 5	0.369	[0.363,0.375]$^{\text {ns}}$	**0**.**669**	[0.662,0.675]$^{\text {ref}}$	0.375	[0.367,0.383]***
Average of means	0.348		0.605		0.269	

Models 1–5 correspond to the models that were saved after training with the fold 1–5 datasets of the cross-validation. The saved models from cross-validation were evaluated on the testset, and the results are based on bootstrapping 100 samples from the testset. Mean and 95% confidence interval (CI) are reported for each base model. Averages of mean values are included. For each performance metric, the best model result is used as a reference for statistical comparison with the remaining models. Indication of results from statistical comparison: *$p<0.05$, **$p<0.01$, ***$p<0.001$, ns = $p>0.05$ (not significant)

### Model Calibration

In order to estimate how well the predictions agree with the true frequencies of the classes, the model ECEs were calculated. The estimation of binned ECE in a multi-class setting comes from a reliability diagram (histogram) with bar heights that represents the average of all class probabilities for the true detections within a probability range (bin). The number of bins within the range of 0 and 1 is either fixed or optimized. Apple’s ml-calibration code from https://github.com/appleml-calibration/blob/main/notebooks/paper_experiments.ipynb was adapted to produce standard reliability diagrams in our study.

### Statistical Analysis

All statistical analyses were performed in Python. Non-parametric statistical tests, specifically the Kruskal-Wallis test for continuous variables and the chi-square test for categorical variables, were applied to the study population characteristics for class-wise comparisons using SciPy Stats functions kruskal and chi2_contingency. A *p*-value of less than 0.05 was considered significant. Post hoc analysis was used to follow up significant differences. For Kruskal-Wallis tests, pairwise group comparisons were performed with Dunn test. The chi-square test of age groups was followed up with additional Spearman’s rank-order correlation coefficient for pairwise comparison between diagnostic classes.

**Table 4:** General classification performance and estimated calibration error (ECE) of the ensemble models shown per architecture

Ensemble model	Balanced accuracy		ROC AUC		ECE	
	Mean	95% CI	Mean	95% CI	Mean	95% CI
**Custom 1**						
Unweighted average	0.568	[0.557,0.579]***	0.741	[0.733,0.749]$^{\text {ns}}$	0.170	[0.163,0.177]$^{\text {ns}}$
Weighted average	**0**.**608**	[0.597,0.618]$^{\text {ref}}$	0.751	[0.743,0.760]$^{\text {ref}}$	**0**.**163**	[0.156,0.171]$^{\text {ref}}$
Majority vote	0.535	[0.525,0.545]***	n/a	n/a		
**Custom 2**						
Unweighted average	0.430	[0.422,0.437]***	0.741	[0.734,0.747]$^{\text {ns}}$	0.151	[0.144,0.157]$^{\text {ns}}$
Weighted average	**0**.**456**	[0.448,0.464]$^{\text {ref}}$	0.744	[0.738,0.751]$^{\text {ref}}$	**0**.**141**	[0.134,0.149]$^{\text {ref}}$
Majority vote	0.422	[0.414,0.430]***	n/a		n/a	
**ResNet-18**						
Unweighted average	**0**.**371**	[0.365,0.376]$^{\text {ref}}$	0.655	[0.649,0.662]*	**0**.**240**	[0.232,0.248]$^{\text {ref}}$
Weighted average	0.369	[0.363,0.375]$^{\text {ns}}$	0.665	[0.659,0.671]$^{\text {ref}}$	0.310	[0.302,0.318]***
Majority vote	0.361	[0.355,0.367]*	n/a		n/a	

The results are based on bootstrapping 100 samples from the test set. Mean and 95% confidence interval (CI) are reported for each ensemble model. The majority vote ensembles are created from hard labels (0,1,2) rather than label probabilities, which does not allow calculation of area under the receiver operating curve (ROC AUC) and ECE. For each performance metric, the best model result is used as a reference for statistical comparison with the remaining models. Indication of results from statistical comparison: *$p<0.05$, **$p<0.01$, ***$p<0.001$, ns = $p>0.05$ (not significant)

For comparison of decision strategies, we bootstrapped (*n*=100) the test population with replacement and calculated the mean and 95% confidence intervals for balanced accuracy, ROC AUC, sensitivity, specificity, precision, and F1 score. Similarly, means with 95% confidence intervals for ECEs were calculated for the decision strategies unweighted and weighted average. Pairwise statistical comparison between the means of the three decision strategies was performed using two-tailed *z*-test for balanced accuracy, ROC AUC and ECE. Aggregated ROC AUCs for the unweighted and weighted average decision strategies were produced based on bootstrapping (*n*=100) the test set with replacement. This was done as one vs. one classification by binarizing the 3 diagnostic classes CN, MCI, and AD, resulting in a total of 300 ROC curves that were averaged to one curve with 95% confidence intervals. All analyses were repeated for base models and ensemble models for all three architectures. The majority vote is based on hard labels rather than probabilities, and therefore, the ECE and ROC AUC were not calculated for this decision strategy.

### Ethical Approval Declarations

This work involved human subjects in its research. The approval of all ethical and experimental procedures and protocols was granted according to “ADNI3 ProtocolVersion3.1” found in the ADNI study documents at https://adni.loni.usc.edu/help-faqs/adni-documentation/ and performed in accordance with GCP guidelines and in full conformity with Regulations for the Protection of Human Subjects of Research codified in 45 CFR Part 46 – Protection of Human Subjects, 21 CFR Part 50 – Protection of Human Subjects, 21 CFR Part 56 - IRBs, and/or the ICHE6, HIPAA, State, and Federal regulations and all other applicable local regulatory requirements and laws. Informed consent was obtained in accordance with US 21 CFR 50.25, the Tri-Council Policy Statement: Ethical Conduct of Research Involving Humans and the Health Canada and ICH Good Clinical Practice. A Data Use Agreement was signed prior to accessing the data.

## Results

### Study Population

Table 2 gives an overview of the characteristics of the total study population and a comparison between the subpopulations of each diagnostic category. The participants included in our study ranged in age from 50 to 90 years, with an average age of 70.83 years. The AD population was older than the healthy population, which is expected as the likelihood of developing AD increases with age. In terms of female-to-male distribution, the population as a whole was fairly balanced with a female ratio of 54.2%, but the CN and AD subpopulations are over- and underrepresented regarding female participants with a 63.4% and 39.7% female proportion, respectively. The distribution of diagnostic categories among subpopulations, based on age and education data split criteria, is included in Table 2. The differences between the CN, MCI and AD populations were statistically significant for age, educational level, cognitive evaluation, and APOE status.

**Fig. 3:**
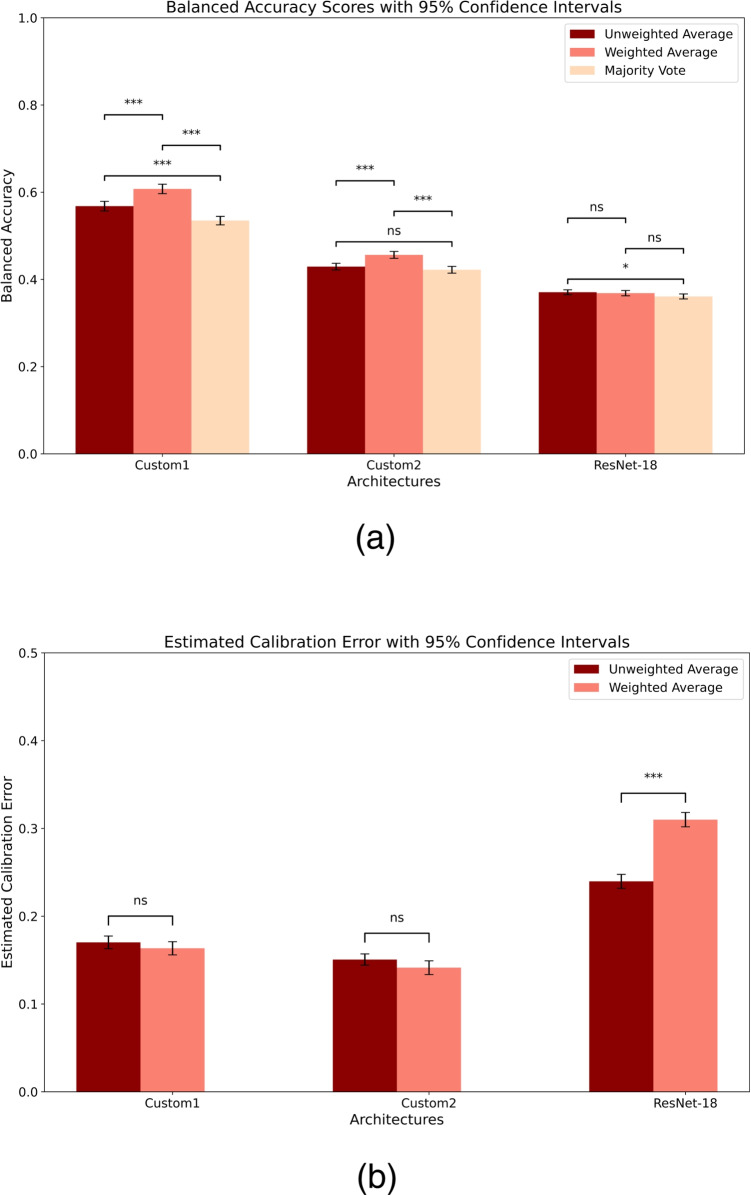
Comparison of detection and calibration performance between decision strategies and model architectures. **a** Balanced accuracy bootstrapped (*n*=100) mean values with 95% confidence interval and **b** estimated calibration error (ECE) bootstrapped (*n*=100) mean values with 95% confidence interval. Results from statistical comparison are indicated: *$p<$0.05, **$p<$0.01, ***$p<$0.001, ns = $p<$0.05 (not significant)

**Fig. 4:**
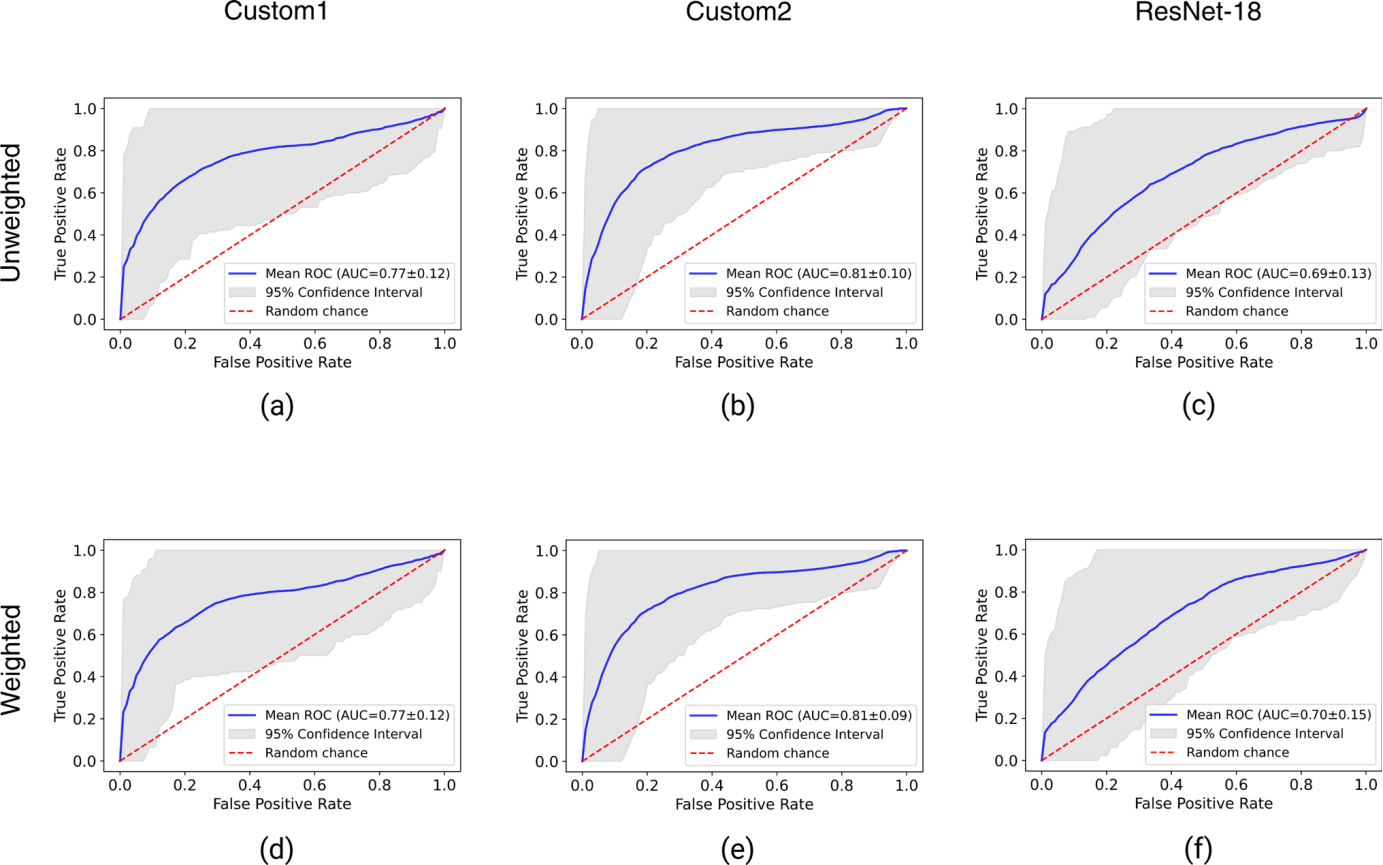
Mean receiver operating curves (ROCs) with 95% confidence intervals for all decision strategies and architectures based on bootstrapping (*n*=100) the test set and binarizing the 3 classes to perform one vs. one classification for cognitively normal (CN), mild cognitive impairment (MCI), and Alzheimer’s disease (AD). The binarized results were aggregated in one ROC curve. The top row (**a**–**c**) shows the results for unweighted average ensemble models, and the bottom row (**d**–**f**) shows weighted average ensemble models

### AD Detection Performance

The individual base model classification performance for all three architectures as evaluated on the test set is presented in Table 3 together with the ECE. The table includes the average of the means of all base models. The performance between base models within each architecture varied, which was also observed for the ECE. However, higher balanced accuracy values did not systematically correspond to low ECE values.

**Table 5:** Additional evaluation metrics sensitivity, specificity, precision, and F1 score for base models of each architecture

Base model	Sensitivity		Specificity		Precision		F1	
	Mean	95% CI	Mean	95% CI	Mean	95% CI	Mean	95% CI
**Custom 1**								
Model 1	0.597	[0.589,0.605]	0.647	[0.640,0.655]	0.636	[0.629,0.643]	0.585	[0.576,0.593]
Model 2	0.652	[0.644,0.660]	0.705	[0.699,0.712]	0.640	[0.631,0.649]	0.635	[0.627,0.644]
Model 3	0.619	[0.610,0.628]	0.732	[0.726,0.738]	0.619	[0.609,0.629]	0.613	[0.604,0.622]
Model 4	0.636	[0.627,0.644]	0.649	[0.641,0.658]	0.622	[0.612,0.631]	0.599	[0.590,0.608]
Model 5	0.672	[0.664,0.681]	0.715	[0.707,0.722]	0.667	[0.658,0.675]	0.658	[0.649,0.667]
Average of means	0.635		0.690		0.637		0.618	
**Custom 2**								
Model 1	0.584	[0.576,0.592]	0.513	[0.504,0.522]	0.678	[0.660,0.696]	0.472	[0.462,0.483]
Model 2	0.618	[0.611,0.626]	0.610	[0.602,0.618]	0.575	[0.563,0.588]	0.553	[0.543,0.562]
Model 3	0.592	[0.584,0.600]	0.686	[0.679,0.693]	0.586	[0.577,0.595]	0.583	[0.574,0.591]
Model 4	0.578	[0.570,0.586]	0.526	[0.518,0.535]	0.503	[0.489,0.517]	0.477	[0.467,0.487]
Model 5	0.609	[0.600,0.618]	0.631	[0.623,0.638]	0.536	[0.525,0.547]	0.556	[0.546,0.567]
Average of means	0.596		0.593		0.590		0.528	
**ResNet-18**								
Model 1	0.545	[0.537,0.554]	0.455	[0.446,0.463]	0.299	[0.290,0.308]	0.386	[0.376,0.395]
Model 2	0.545	[0.537,0.554]	0.455	[0.446,0.463]	0.299	[0.290,0.308]	0.386	[0.376,0.395]
Model 3	0.533	[0.524,0.541]	0.497	[0.487,0.506]	0.456	[0.442,0.471]	0.434	[0.424,0.443]
Model 4	0.523	[0.515,0.532]	0.487	[0.479,0.495]	0.408	[0.397,0.420]	0.428	[0.418,0.438]
Model 5	0.558	[0.550,0.567]	0.500	[0.490,0.509]	0.531	[0.513,0.549]	0.450	[0.440,0.460]
Average of means	0.541		0.479		0.399		0.421	

Models 1–5 correspond to the models that were saved after training with the fold 1–5 datasets of the cross-validation. The saved models from cross-validation were evaluated on the testset, and the results are reported here as mean values with a 95% confidence interval (CI) after bootstrapping 100 samples of the testset. Averages of mean values of the five base models are included

The results from pairwise statistical comparison for balanced accuracy, ROC AUC, and ECE are presented in Tables 3 and 4. In the tables, the statistical analysis is presented pairwise between the best model as a reference compared one-to-one with the other models, while Fig. 3 shows all pairwise comparisons for balanced accuracy and ECE. The results of comparing the detection performance of the decision strategies are visualized in Fig. 3a, where the bars represent the mean balanced accuracy after bootstrapping, and the error bars represent the 95% confidence intervals (CI). For the Custom1 architecture, the results show a difference between the decision strategies with respect to balanced accuracy. This also applies to the Custom2 architecture for the comparison between weighted average and majority vote, and between weighted average and unweighted average, but not between unweighted average and majority vote. Regarding the ResNet-18 architecture, only the result from comparing unweighted average and majority vote suggests a difference in decision strategies with respect to balanced accuracy. The visual presentation in Fig. 3b shows that although the weighted average strategy has a lower calibration error, it is not significantly different from the unweighted average strategy in terms of ECE for two of the three architectures. Unlike the ECE results for the ResNet-18 architecture, there is a significant difference both between the decision strategies and also compared to the two other architectures.

Figure 4 visualizes the detection performance in terms of aggregated mean receiver operating curves (ROC) from bootstrapping (n=100) the test set and binarizing the classes cognitively normal (CN), mild cognitive impairment (MCI), and Alzheimer’s disease (AD) to allow one vs. one classification. The broad 95% confidence intervals show the variation between the bootstrapped datasets and were probably affected by class imbalance. The conditions were the same between architectures and decision strategies, allowing for comparison. The two customized architectures performed better than ResNet-18, where Custom2 had the highest area under the receiver operating curve (ROC AUC) of 0.81 compared to Custom1 with a ROC AUC of 0.77. In terms of ROC AUC, the weighted average and unweighted average strategies performed equally.

**Table 6:** Additional evaluation metrics sensitivity, specificity, precision, and F1 score for ensemble models of each architecture

Ensemble model	Sensitivity		Specificity		Precision		F1	
	Mean	95% CI	Mean	95% CI	Mean	95% CI	Mean	95% CI
**Custom 1**								
Unweighted average	0.656	[0.648,0.664]	0.692	[0.684,0.701]	0.648	[0.639,0.658]	0.638	[0.629,0.647]
Weighted average	0.680	[0.672,0.688]	0.720	[0.711,0.727]	0.675	[0.666, 0.684]	0.665	[0.656,0.674]
Majority vote	0.681	[0.673,0.689]	0.731	[0.724,0.737]	0.663	[0.654,0.672]	0.659	[0.650,0.667]
**Custom 2**								
Unweighted average	0.618	[0.610,0.626]	0.595	[0.588,0.602]	0.600	[0.584,0.615]	0.549	[0.540,0.559]
Weighted average	0.641	[0.632,0.649]	0.644	[0.637,0.651]	0.608	[0.595,0.622]	0.590	[0.580,0.600]
Majority vote	0.610	[0.602,0.618]	0.606	[0.598,0.613]	0.559	[0.546,0.573]	0.542	[0.532,0.552]
**ResNet-18**								
Unweighted average	0.566	[0.558,0.574]	0.499	[0.489,0.508]	0.564	[0.543,0.586]	0.445	[0.435,0.454]
Weighted average	0.558	[0.550,0.567]	0.500	[0.490,0.509]	0.531	[0.513,0.549]	0.450	[0.440,0.460]
Majority vote	0.550	[0.542,0.559]	0.481	[0.471,0.491]	0.525	[0.506,0.544]	0.434	[0.425,0.444]

The results are reported as mean values with a 95% confidence interval (CI) after bootstrapping 100 samples of the testset

**Fig. 5:**
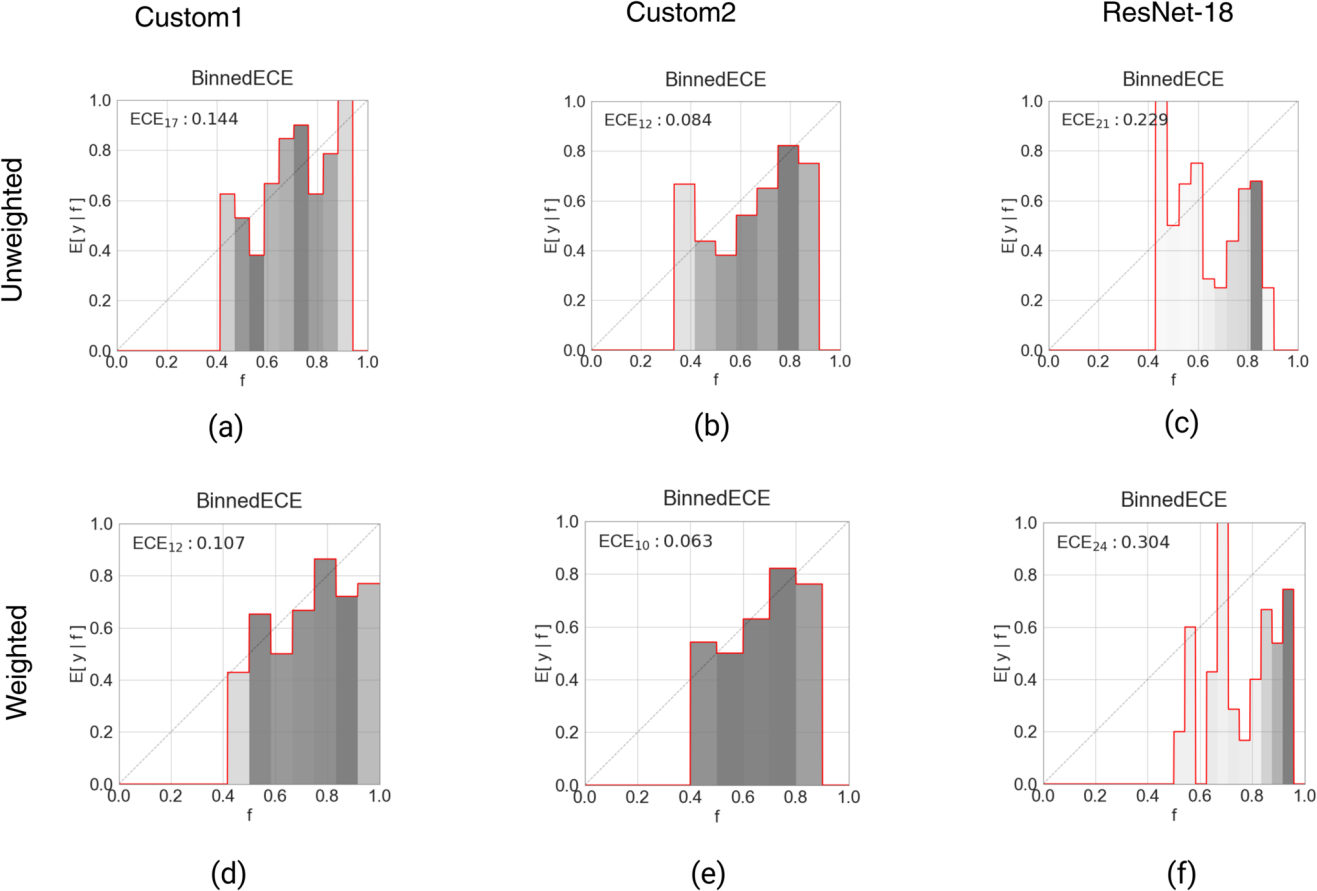
Examples of reliability diagrams showing the Expected Calibration Error (ECE) for ensemble models of all three architectures based on the original test set without bootstrapping. The top row (**a**–**c**) shows ensemble models with the unweighted averages decision strategy, and the bottom row (**d**–**f**) shows ensemble models with the weighted average strategy. The majority vote strategy is not shown here as it is based on hard labels, and ECE requires information about class probabilities

The performance of the ensemble models using three different decision strategies is presented in Table 4. The ensemble models with averaging decision strategies achieved the highest performance based on balanced accuracy, which considers the class imbalance contrary to the commonly reported standard accuracy. For the two customized VGG-like architectures, the results suggest that the weighted average strategy increased the prediction ability, while the ResNet-18 architecture performed moderately better with the unweighted average strategy. Concentrating on detection performance results only, the Custom1 architecture seemed to provide the best detection between CN, MCI, and AD. For completeness, sensitivity (recall), specificity, precision, and F1 score results are reported for base models and ensemble models in Tables 5 and 6, respectively.

### Calibration Error

The expected calibration errors (ECE) after bootstrapping (*n*=100) are reported in Tables 3 and 4 for base models and ensemble models, respectively. The reliability diagrams in Fig. 5 were plotted for the original test population before bootstrapping. The difference between the bootstrapped ECE means between the unweighted and the weighted average decision strategy is visualized per architecture in Fig. 3b. The results were as follows: Custom1, 0.007 (95% confidence interval (CI), $-$0.014, 0.027); Custom2, 0.009 (95% CI, $-$0.011, 0.029); ResNet-18, 0.070 (95% CI, 0.048, 0.093). These results suggest that there was a significant difference in the degree of miscalibration between the unweighted and weighted average decision strategies for the ResNet-18 model. For the other two architectures, the results suggest that there was no significant difference between choosing the unweighted average and the weighted average.

### Weights for the Weighted Average Decision Strategy

Table 7 lists the balanced accuracy values for each fold and all architectures derived from cross-validation training, the assigned low to high ranks based on low to high balanced accuracy, and the resulting weights that were used to prioritize the contributions of each base model in the weighted average ensembles. There was no clear ranking pattern between the architectures, and consequently, the weight assigned to each base model varies between the architectures.

## Discussion

Structural brain MRI is a widely used diagnostic tool in the clinic today and has an important supportive role both in the AD diagnostic pathway and in AD treatment monitoring. However, today’s clinical use of MRI has limited impact in terms of intervention early in the AD trajectory. Our study explored the relationship between AD detection performance and model calibration for parallel ensemble methods with the aim of improving the clinical translatability of AD detection with structural brain MRI.

In our work, we found that the best detection performance corresponded to the best model calibration for ensemble models across all architectures, based on balanced accuracy and ECE. However, the same was not true for the base models. For the two customized architectures, the weighted average ensemble model had the best balanced accuracy, but the calibration error was not significantly different from the unweighted average ensemble model. Both averaging strategies had a significant advantage over the majority vote strategy in terms of balanced accuracy. For the ResNet-18 architecture, we did not find evidence of differences in balanced accuracy between the decision strategies, but the weighted average had a significantly larger calibration error than the unweighted average.

**Table 7:** Weights calculated for the weighted average decision strategy

	Fold 1	Fold 2	Fold 3	Fold 4	Fold 5
**Custom1**					
Balanced accuracy	0.485	0.704	0.635	0.734	0.783
Rank	1	3	2	4	5
Weights	0.014	0.159	0.067	0.290	0.470
**Custom2**					
Balanced accuracy	0.527	0.729	0.728	0.526	0.600
Rank	2	5	4	1	3
Weights	0.063	0.470	0.301	0.016	0.151
**ResNet-18**					
Balanced accuracy	0.333	0.328	0.471	0.582	0.609
Rank	2	1	3	4	5
Weights	0.060	0.015	0.153	0.298	0.475

The balanced accuracy values that were used to determine the weight factors were calculated from the validation test results of the cross-validation. The weight factors in the table have rounded values, but the sum of the applied unrounded weight factor values is 1

Ju et al. [45] discuss the consequences of overconfident models, stating that overconfident base models will dominate unweighted average ensembles and that higher degrees of overconfidence contribute to unweighted average ensembles being comparable to majority vote ensembles. Another challenge with the unweighted average strategy is that the combination of base models with prediction probabilities at the high and low ends of the scale evens out the extreme values. This could potentially reduce the influence of base models with higher predictability power. The majority vote strategy has the risk of being imprecise with respect to its hard cut-off at a threshold value that is often not scientifically justified. In the multi-class setting with softmax class probability, the majority vote favors the winning class regardless of the similar class probability of another class. Weighted average strategies enable the inclusion of prior knowledge in the determination of weights, which can favor stronger performing base models over weaker ones, with the risk of including bias. The detection results in our study suggest a preference for averaging ensembles over majority vote, but the calibration results did not show a clear preference between unweighted and weighted average. With the ECE calibration method, we were unable to compare calibration differences between the averaging strategies and the majority vote strategy.

Our analysis of consistency across architectures showed a similar pattern for the two customized models, which are VGG-based, and that the ResNet-18 model responded differently from the other two under the same testing conditions. The ResNet-18 model performed inferiorly to the other two models and also had the highest calibration error. It could be questioned whether the higher complexity of the deeper ResNet-18 architecture compared to the two custom networks exceeded the requirements of the task. In particular, Wen et al. [46] concluded that a reduction in convolutional blocks was advantageous to detection performance when they optimized a CNN for AD detection. Their proposed architecture was based on a review of the literature for neural networks used with structural MRI and experimentation with a number of combinations of hyperparameters. Furthermore, Guo et al. [34] showed increased model calibration for factors that have contributed to a significant increase in classification performance for convolutional neural networks compared to earlier machine learning models and simpler neural networks. Increased layer depth and width, batch normalization, and less weight decay are examples of such factors. The VGG-based customized models in our work have the same layer depth and differ mainly in the number of filters, kernel sizes, and choice of normalization method. However, the Custom2 model had more to gain from the ensemble than the Custom1 model in terms of reducing miscalibration.

There are several studies that use brain structural MRI with parallel ensembles to detect AD [18, 22, 23, 27–31], but a major shortcoming of these studies is that they do not quantify the calibration error of their models. For AD detection from 3D structural MRI, Dang et al. [37] analyzed both the calibration error and the detection performance with the purpose of fairness evaluation. Without the use of ensemble models, they applied a CNN architecture similar to the two custom architectures used in our work. They observed a correspondence between performance biases, using false negative rates and false positive rates, and calibration biases using ECE, Brier score, and calibration curves. However, Dang et al. [37] point out that imbalance between the subgroup classes could have affected the ECE results. Rajaraman et al. [12] found that model calibration and performance react to changes in class balance and the ratio of positive abnormal samples. In their work, they applied recalibration to decrease the calibration error using a variety of calibration methods. In binary experiments with fundus images and chest X-ray images, they showed a significant improvement of model accuracy for calibrated probabilities compared to uncalibrated probabilities when the standard threshold of 0.5 was applied. However, the use of optimal thresholds based on precision-recall curves resulted in equal performance for uncalibrated and calibrated probabilities.

## Conclusion

We investigated the consistency of AD detection and calibration across different architectures and three different common decision strategies using a publicly available dataset as input in a typical DL scenario. We applied a standard classic neural network as a benchmark for two custom neural networks previously proven useful in AD detection. We observed the interplay between detection performance and model calibration for ensemble models trained with small imbalanced datasets applied in a multi-class setting of AD detection. The results indicated architecture dependence in terms of the best ensemble model decision strategy, but a consistency across architectures concerning the association of the best detection performance with the lowest calibration error when using ensemble methods. The results suggest that parallel ensemble models have the ability to increase the confidence in AD detection to enable more reliable decision-making in the clinic. The work confirmed that parallel ensemble methods provide a simple approach to gain much value from little expense.

There are several limitations in our study. Compared to the related work listed in Table 1, the detection performance does not match that of the other studies. Comparison between studies is difficult due to the use of different datasets, dataset sizes and balances, different network architectures, and different evaluation metrics. However, this could be attributed to our restrictions of imposing equal conditions when training the different architectures. This was done to be able to observe tendencies that are independent of the architecture. Consequently, the training did not benefit from independent hyperparameter optimization for each architecture, and the base model detection performance would have suffered from this. Furthermore, DL detection studies should ideally include an external validation set. However, the main motivation for our work was to observe the effect of the different ensemble decision strategies on classification performance and calibration error, and the relationship between detection performance and calibration error for parallel ensemble models. A further limitation is that the scope of this study did not include an analysis of how variation of the degrees of class imbalance could affect the relationship between detection performance and calibration error. Furthermore, we were unable to compare the miscalibration of the majority vote strategy with the other decision strategies due to metrics that require predicted probabilities. We limited our work to three decision strategies that cover the most used decision strategies in the literature and did not explore alternative weighting strategies or variations such as weighted majority voting.

Future work could consider alternative variations of common decision strategies. Uncertainty quantification of individual predictions and application of calibration methods to adjust prediction probabilities are other research areas to pursue. Finally, the relationship between detection performance and calibration error should be addressed for subgroups of the study population based on relevant characteristics of AD.

## Data Availability

Data from the ADNI study are available upon application and acceptance of the data use agreement and are shared with approved researchers through the LONI Image and Data Archive (IDA), a secure repository of research data.

## Materials Availability

Materials availability A list of participants that were used in our study is available on the GitHub repository https://github.com/SolveigCodes/AI_ensembles_for_AD.

## Code Availability

The code for training and testing neural networks has been made available on the GitHub repository https://github.com/SolveigCodes/AI_ensembles_for_AD.
